# Structural Model of Biomedical and Contextual Factors Predicting In-Hospital Mortality due to Heart Failure

**DOI:** 10.3390/jpm13060995

**Published:** 2023-06-13

**Authors:** Juan Manuel García-Torrecillas, María Carmen Lea-Pereira, Enrique Alonso-Morillejo, Emilio Moreno-Millán, Jesús de la Fuente-Arias

**Affiliations:** 1 Emergency and Research Unit, Torrecárdenas University Hospital, 04009 Almería, Spain; 2 CIBER de Epidemiología y Salud Pública (CIBERESP), 28029 Madrid, Spain; 3 Instituto de Investigación Biosanitaria Ibs, 18012 Granada, Spain; 4 Hospital de Poniente, El Ejido, 04700 Almería, Spain; maru31es@yahoo.es; 5 School of Psychology, University of Almería, 04120 Almería, Spain; ealonso@ual.es; 6 Equipo de Investigación SEJ-581, Departamento de Economía Aplicada, Universidad de Almería, 04120 Almería, Spain; emormill@gmail.com; 7 School of Education and Psychology, University of Navarra, 31009 Pamplona, Spain; jdlfuente@unav.es; 8 School of Education and Psychology, University of Almería, 04120 Almería, Spain

**Keywords:** SEM analysis, heart failure, biomedical factor, in-hospital factors, epidemiology, mortality

## Abstract

*Background*: Among the clinical predictors of a heart failure (HF) prognosis, different personal factors have been established in previous research, mainly age, gender, anemia, renal insufficiency and diabetes, as well as mediators (pulmonary embolism, hypertension, chronic obstructive pulmonary disease (COPD), arrhythmias and dyslipidemia). We do not know the role played by contextual and individual factors in the prediction of in-hospital mortality. *Methods*: The present study has added hospital and management factors (year, type of hospital, length of stay, number of diagnoses and procedures, and readmissions) in predicting exitus to establish a structural predictive model. The project was approved by the Ethics Committee of the province of Almeria. *Results*: A total of 529,606 subjects participated, through databases of the Spanish National Health System. A predictive model was constructed using correlation analysis (SPSS 24.0) and structural equation models (SEM) analysis (AMOS 20.0) that met the appropriate statistical values (chi-square, usually fit indices and the root-mean-square error approximation) which met the criteria of statistical significance. Individual factors, such as age, gender and chronic obstructive pulmonary disease, were found to positively predict mortality risk. Isolated contextual factors (hospitals with a greater number of beds, especially, and also the number of procedures performed, which negatively predicted the risk of death. *Conclusions*: It was, therefore, possible to introduce contextual variables to explain the behavior of mortality in patients with HF. The size or level of large hospital complexes, as well as procedural effort, are key contextual variables in estimating the risk of mortality in HF.

## 1. Introduction

Heart failure (HF) is a syndromic process with high prevalence and rising incidence, especially in Western countries. In Spain the prevalence was, in 2019, 1.89 (CI95% 1.70–2.08) with an incidence rate of 2.78 new cases per 1000 people and year [1], having projected a 30% increase in 2035 [2], mainly due to ageing, although in France it has increased especially among young adults (36–59 years) [3]. It is currently the most frequent cause of hospital admission in patients over age 65 and constitutes 5% of total hospital admissions in Spain during recent years. The most common hospital access for patients with decompensated HF is through the emergency department [4], and from this department the stabilized patient may be discharged to home, or admitted to a hospital ward or short-stay unit [5]. In-hospital mortality for decompensated HF worldwide was about 9–13% [6]. In our country, studies such as Escobar et al. focused on hospitalized patients of internal medicine and showed in-hospital mortality at 9.5%; these percentages may significantly increase when a geriatric population is being considered—potentially as high as 11% mortality [7]. In Spain, hospital mortality during admissions for acute HF is variable and constitutes a major problem from the point of view of public health and cost efficiency, putting a strain on healthcare expenditures [1,2,8].

On one hand, we must consider that the improved care and survival of ischemic heart disease in recent years constitute true breakthroughs in cardiology, but increased survival of ischemic heart disease results in very high rates of HF with this aetiology, not to mention the increase that comes along with the unrelenting aging of the population. It is important to keep in mind that HF is not a unique nosological process, but it is one step in the physiopathological route of multiple morbid entities and pathologies, resulting in its extreme prevalence.

To date, there are only two epidemiological, populational studies of HF in Spain (Cortina et al.) [9], in the region of Asturias and the Price Study nationwide [10]. In-hospital estimates of mortality are more reliable than populational estimates, and, despite the high levels of mortality in HF, there are no explanatory models with a predictive component that are founded on large clinical-administrative databases such as the Basic Minimum Data Set (BMDS). The BMDS is a clinical-administrative database of obligatory use in all the hospitals of the Spanish National Health System that is complete with data from the clinical history of the patients. From these databases, different categories can be established according to the “diagnosis-related groups” (DRGs). The DRGs are a system of classification of patients that allows the relating of the casuistry of treated patients with their cost. This system allows classification into categories (DRGs) with the isoconsumption of resources.

Nor is there any approach based on structural equations methodology so that we may gain knowledge of how variables at different levels interact to influence mortality in these patients.

The main objective of this study was to determine whether it is feasible to develop a structural model to understand the functioning of direct and intermediate factors associated with in-hospital mortality due to decompensated HF in Spain. A secondary objective was to determine the role of 30-day readmission as a contextually dependent variable and its relationship with in-hospital mortality.

## 2. Materials and Methods

### 2.1. Participants

*Design*: A retrospective cohort study was designed using analytical observation of all hospital stays for HF during the period 2008–2012. Two diagnosis-related groups (DRGs) were studied: HF or shock without comorbidities (DRG 127) and complex HF (DRG 544), with comorbidities. *Geographic scope.* The study was developed within the sphere of the Spanish National Healthcare System, which has a decentralized structure in 17 autonomous or regional healthcare systems whose information is centrally collected in the Ministry of Health and Consumerism. Each of the autonomous systems has its own structure with Basic Healthcare Areas that are in turn grouped into Primary Care Districts and Hospitals; in our case, we have dealt exclusively with patients’ hospitalization episodes, and walk-in patients were excluded.

### 2.2. Source of Information, Sample and Case Selection

The source of information was Spain’s Basic Minimum Data Set (BMDS) at discharge, made available by the Ministry of Health, Consumerism and Social Policies [11]. This database contains information from more than 300 hospitals in Spain and is built up from the information contained in the medical records. Information about each hospitalization is sent by the different hospitals to the Ministry of Health, where all the information is centralized. In each episode, administrative and clinical data are collected, especially the diagnoses (the main one and up to 13 secondary ones), as well as up to 20 categories of procedures performed on the patients. A total of 529,606 hospital stays were analyzed, the total of all hospital stays from the period studied. Diagnostic and procedural coding was carried out using the International Classification of Diseases, Ninth Revision, and Clinical Modification (ICD-9-CM). The selection criteria consisted of extracting the patient stays that were discharged under the DRGs 127 and 544, after applying the AP-DRG classifier, version 21. DRG 127 accounted for 57.4% of the hospital stays (304,405 stays) and the remaining 42.6% was accounted for by DRG 544 (225,561).

### 2.3. Variables

The main dependent variables were mortality, an individual-based variable, and readmission, a context-based variable. The variables studied were sociodemographic (age, gender and healthcare region) and clinical (number of diagnoses at discharge “NDX” and the number of procedures at discharge “NPR”). NDX was considered a proxy for comorbidity or disease burden while NPR was the proxy for therapeutic effort and procedural complexity. In addition, we analyzed the type of admission (urgent vs. scheduled), as well as hospital management variables (length of stay, hospital group and readmission). The variables that, according to the evidence, are associated with a greater risk of morbimortality from cardiovascular causes (diabetes, hypertension (HTA), dyslipemia and obesity, especially) were considered. Other variables such as the existence of arrhythmias (ARR), renal insufficiency (RI) and anemia, among others, were also considered to define the patient’s comorbidities profile.

Only mortality that took place during hospitalization was taken into account. Readmission was counted when it occurred within 30 days after discharge, as long as it was classified under the same DRGs and did not occur in a different calendar year. Unplanned hospital admissions included any order for urgent hospital admission regardless of whether the patient came to the hospital via the emergency department or through other channels.

### 2.4. Instruments and Procedure

#### Bioethics Committees

The appropriate confidentiality and good practices documents, as approved by the Ministry of Health and Social Policies and in accordance with the legislation in force, were signed. Subsequent use and transmission of data, from the Ministry to the researchers, were anonymous and untraceable. The present investigation was evaluated and approved by the Provincial Research Ethics Committee (Ref. 72/2018).

### 2.5. Data Analysis

#### 2.5.1. Data Analysis Strategy

In order to address our main objective, and detect the factors that are associated with in-hospital mortality for HF, we began with the hypothesis that each individual variable in the linear model (age, gender and the main individual comorbidities) would have a statistically significant effect on the intermediate variables in the model (length of stay, NDX, NPR and context variables such as year and hospital group); these intermediate variables would, in turn, have such an effect on the two main dependent variables, that is, mortality in the individual dimension and readmissions in the contextual dimension.

#### 2.5.2. Variables and Analysis Schema

The analysis schema defined two axes for the study of relations and associations between variables, as seen in Table 1. On one hand, variables were analyzed according to two large dimensions of each episode: variables in the individual dimension and variables in the contextual dimension. The context variables were identified as year, hospital group, length of stay, NDX and NPR, as well as readmissions, which was considered the dependent, context variable; the remaining variables were considered variables characteristic of the individual (Table 1). On the other hand, our second axis of analysis classified variables as predictor variables, mediating/process variables or outcome/criteria variables regardless of the dimension to which they belonged.

As can be observed in the second part of Table 1, the variables are grouped into two distinct columns: individual variables and context-specific variables. For each of them, the way they are measured is indicated (percentages, yes/no values, specific categories in the case of polytomous variables and exact values in the case of quantitative variables).

Sociodemographic information was obtained from the variables year, age, gender and autonomous region (Spain). Administrative-type elements were assessed through the variables length of stay, 30-day readmission in the same DRG, type of admission (emergency vs. scheduled) and type of discharge (alive vs. exitus). Readmission was counted when it occurred within 30 days after discharge and in the same DRG. From a clinical point of view, we used the NDX as a proxy variable for the patient’s comorbidity, and the NPR to estimate the procedural complexity and therapeutic effort of each episode and the main clinical comorbidities associated with HF episodes.

### 2.6. Statistical Analysis

For the statistical analysis, variables were treated as follows, according to the dimension being analyzed: (1) first, the initial variables were the predictor variables (PV), and the process and outcome variables were mediating variables (MV); (2) second, the process variables were the predictor and the outcome variables exitus (death) and readmission were the criteria variables (CV), according to the dimension of analysis.

Two types of analysis were carried out in order to determine the variables to be included in the structural linear model. First, bivariate analysis was carried out; Student’s *t* test was used to test the equality of means hypothesis for independent samples or analysis of variance. In cases where they could not be applied, the Mann–Whitney or Kruskal–Wallis nonparametric test was applied, as appropriate (see Table S1 of the Supplementary Material). The chi-square test was used for comparison of qualitative variables. Relationships between quantitative variables were determined through Pearson correlations (see Supplementary Material). Second, uni- and multi-variate inferential analysis was carried out between the variables established in the rational model. Inferential statistical analyses (multivariate analysis, MANOVAs) were carried out using SPSS (v. 23.0) for Windows. A selection of the most relevant results is shown in this text.

Once the variables were identified, a structural equations model was finally developed. AMOS (v. 23.0) for Windows was used for structural validity analysis and for constructing the structural prediction model—specifically, in verification of the structural linear prediction hypothesis (path analysis). We attempted to replicate the same analysis scheme as in a previous paper (de la Fuente et al. 2019 [12], with a different sample and problem. In model 1, we tested the relationships of the 17 variables, without constructing second-level latent variables. In models 2 and 3, predictive relationships between the latent variables or defined dimensions were tested, with different predictions between them. In model 2, predictive relationships were established that were less significant. Finally, model 3 showed the most robust ones, with acceptable overall significant effects.

To interpret the confirmatory factor analysis (CFA) and the structural equation model (SEM) fit, we assessed model fit by first examining the comparative fit index (CFI), normed fit index (NFI), incremental fit index (IFI), relative fit index (RFI) and the root-mean-square error of approximation (RMSEA). Sample size adequacy was checked using the Hoelter index (Tabachnick&Fidell, 2001). The analyses were conducted using the AMOS Program (IBM, USA). CFI values equal to or more than 0.90 were taken to indicate an acceptable and close fit to the data (McDonald & Marsh, 1990). RMSEA values equal to or below 0.05 and 0.08 were taken to indicate close and acceptable levels of fit, respectively (Jöreskog&Sörbom, 1993 [13]. Keith (2006) [14] proposed the following research benchmarks for direct effects (direct linear prediction between one variable and another) in the form of beta coefficients: less than 0.05 is considered too small to be meaningful, above 0.05 is small but meaningful, above 0.10 is moderate and above 0.25 is large. For indirect effects (linear prediction between one variable, through another), we used Kenny’s (2012) [15] definition of an indirect effect as the product of two effects; using Keith’s benchmarks above, we propose an educationally meaningful small indirect effect = 0.003, moderate = 0.01 and large = 0.06. Direct values refer to the direct linear prediction of one variable over another. Indirect effects refer to indirect linear prediction, or of a variable through another intermediate. This can occur between latent variables or between a latent variable and another observable through another latent.

## 3. Results

### 3.1. Descriptive Analysis

We analyzed 529,606 hospitalization episodes under DRG 127 (noncomplex HF) and DRG 544 (complex HF with comorbidity), from the years 2008–2012. The mean age of the patients was 79.02 years (SD 10.64). Patients were hospitalized for a mean length of stay of 7.28 days (SD 4.50) and were discharged with 9.31 recorded diagnoses (SD 2.89). They were submitted to 2.61 (SD 2.62) procedures during hospitalization.

The sample included 296,013 female patients (55.9%). Of the total sample, 95.6% of the hospital admissions were unplanned (urgent) and 10.2% (53,862 patients) died during their hospitalization. The global analysis showed that 17.2% of the episodes involved patients who were experiencing readmission to hospital for HF.

Considering the ICD9-CM codes used, the most prevalent were 428.0 (55.8%), 428.1 (12.2%), 402.9 (9.3%) and 428.23 (1%), with the remaining codes associated with HF accounting for 21.7%.

### 3.2. Bivariate Association

Many significant relations of bivariate association appeared between the different variables (*p* < 0.001). These associative relationships served to establish the later model of predictive linear relations. Most of these associations are physiopathologically coherent and have a low level of correlation, but they are associations that allow the establishment of the second-level model. In this sense, the positive association of gender (women) with hypertension and negative with the existence of COPD is noteworthy. Similarly, the NDX was positively associated with the existence of diabetes, the year in which the coding was done (“year”), HTA, dyslipemia and length of stay. Finally, mortality (“exitus”) was associated with age, renal failure and correlated with short stays and negative correlation. See Table 2.

### 3.3. Linear Relations: Structural Prediction

The results of structural analysis or pathway analysis (SEM) showed an acceptable model of relationship between variables. Three relationship models were tested with 17 variables, but only the third showed adequate indices. The relationship parameters of both models are presented below (Table 3).

### 3.4. Standardized Direct Effects

**Figure 1:**
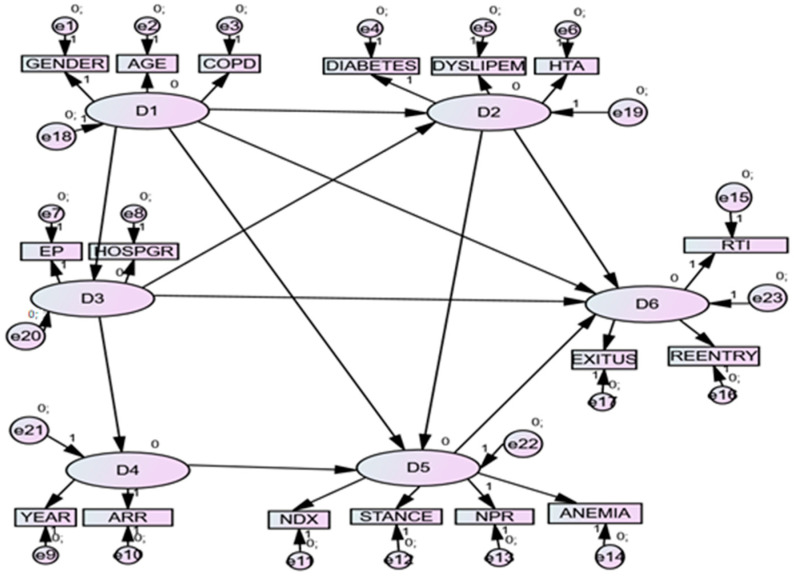
Structural model of relationships of second-level with factors. *Note*. D1 = PRESAGE FACTORS OF PATIENS; D2 = PROCESS FACTORS OF PATTIENTS: ASSOCIATED PATHOLOGIES; D3 = PRESAGE FACTOR OF CONTEXT: TYPE OF HOSPITAL; D4 = PROCESS OF CONTEXT: YEARS; D5= PROCESS OF CONTEXT: INTERVENTIONS; D6 = PRODUCT OF PATIENTS: DEATH; AGE = age; GEND = gender; ANEM = anemia; DIAB = diabetes; HTA = arterial hypertension; ARR = arrhythmias; COPD = chronic obstructive pulmonary disease; YEAR = year; HOSPGR = hospital group; TEP = pulmonary thromboembolism; STAY = length of stay; NDX = number of diagnoses; NPR = number of procedures; READ = readmission. EXITUS = death. RTI = renal insufficiency.

The second-order model showed significant predictions, simpler than the first, as the factors were grouped in latent dimensions. See Table 4 and Figure 1.

The D1 (PRESAGE FACTORS OF PATIENTS) dimension, made up of Age, Gender and Anemia factors, appeared as a positive predictor of D2 (PROCESS FACTORS OF PATIENTS: ASSOCIATED PATHOLOGIES) and D3 (PRESAGE FACTOR OF CONTEXT: TYPE OF HOSPITAL), negative from D5 (PROCESS OF CONTEXT: INTERVENTIONS) and positive from D6 (PRODUCT OF PATIENTS: DEATH).

The D2 dimension (PROCESS FACTORS OF PATIENTS: ASSOCIATED PATHOLOGIES), made up of diabetes, dyslipidemia and HTA, positively predicted D5 (PROCESS OF CONTEXT: INTERVENTIONS), and negatively predicted D6 (PRODUCT OF PATIENTS: DEATH).

The D3 (PRESAGE FACTOR OF CONTEXT: TYPE OF HOSPITAL) dimension, made up of hospital group and EP, positively predicted D2 (PROCESS FACTORS OF PATIENTS: ASSOCIATED PATHOLOGIES) and D4 (PROCESS OF CONTEXT: YEARS), but negatively predicted D6 (PRODUCT OF PATIENTS: DEATH) with great force.

The D4 (PROCESS OF CONTEXT: YEARS) dimension, made up of the Year and ARR, positively predicted D5 (PROCESS OF CONTEXT: INTERVENTIONS).

The dimension D5 (PROCESS OF CONTEXT: INTERVENTIONS), forced by NDX, length of stance, NPR and anemia, positively predicted D6 (PRODUCT OF PATIENTS: DEATH).

### 3.5. Standardized Indirect Effects

The model also revealed the existence of multiple indirect predictions among the variables. The dimension D1 (PRESAGE FACTORS OF PATIENTS), made up of gender and age, has a positive influence on dimensions D2, D4 and D5, and on their factors of diabetes, dyslipemia, HTA, EP, hospital group, year and ARR, as well as negative in D4 (IR, NDX, length of stance, NPR) and D6 (exitus).

The second effect of interest is related to the fact that, while D2 (PROCESS FACTORS OF PATIENTS: ASSOCIATED PATHOLOGIES) appeared as a positive predictor of D5 (PROCESS OF CONTEXT: INTERVENTIONS) and D6 (PRODUCT OF PATIENTS: DEATH), D3 (FACTOR OF CONTEXT: TYPE OF HOSPITAL) was a positive predictor.

The dimension D4 (PROCESS OF CONTEXT: YEARS) appeared as a negative predictor of D6 (PRODUCT OF PATIENTS: DEATH). Additionally, D5 (PROCESS OF CONTEXT: INTERVENTIONS) appeared as an indirect predictor of D and factors (PRODUCT OF PATIENTS: DEATH. See Table 5.

## 4. Discussion

The present study confirmed the role of individual and personal factors in the risk of mortality due to HF. At the same time, and for the first time, it has been documented that contextual factors are key to this risk estimation. It was possible to introduce contextual factors in the elaboration of an explanatory and predictive model of mortality and, given the heterogeneity in the characteristics and equipment of the hospital centers, we believe that this represents a great advance in our knowledge of the risk of mortality due to HF. More specifically, the size of the hospital complexes and the procedural effort was key to the model obtained.

### 4.1. First Level Model

In-hospital mortality, as an undeniably individual variable, is represented and predicted by a number of elements that were already known, and by others that require further explanation; at the same time, mortality is not only produced as a direct effect, but is influenced by mediating variables pertaining to the individual and contextual spheres (Supplementary Material, Model S1).

Thus, age is a direct predictor of mortality in HF; this being plainly understood and present in certain statistical models of mortality [16,17,18], and it supports the plausibility of the present model. Similarly, we observed that age predicted greater prevalences of HTN and ARR, as would be expected from the physiopathological point of view [19]. We feel that age is an ideal variable for testing the model, inasmuch as it fits the biological plausibility and well-understood natural history of HF.

The female sex was statistically associated with an increased risk of mortality; we believe this to be true because the age sectors affected by HF are predominantly female. Prevalence of mortality is systematically higher when we work with age groups where there is a higher death rate for men, and the living population is primarily female—a well-known phenomenon for many entities in epidemiology and demography. Finally, we would emphasize that the female gender also predicts greater mortality by indirect means, through ARR, most often found in elderly patients who, as we have noted, are mostly women.

Patients who suffer from renal insufficiency also present a higher prevalence of mortality, as is documented in prior evidence [19,20,21,22]. Anemia is present in more than half of HF patients, especially if there is renal insufficiency, and this is in relation to the degree of both kidney and HF. The role of anemia in the literature is variable; it appears as an intermediate variable not systematically associated with a long-term prognosis in the evidence reported by other authors [23,24,25,26,27], although certain population registries have associated low levels of hemoglobin with a poorer long-term prognosis [25]. Our results support anemia as more of a marker associated with gender, renal insufficiency and probably poorer clinical conditions than as a variable directly associated with mortality, in agreement with what other authors have suggested [21,22,23,24,25,26,27].

PE, a classic, powerful predictor of mortality in HF, besides being a cause of death in its most acute form [28,29], is marked indelibly as a predictor of mortality in our structural model. Length of stay negatively predicts mortality and this fits into the logic defined in prior studies [30]: the most seriously ill patients are those who die shortly after their hospital admission; therefore, the subgroup of patients with very short hospital stays normally presents high mortality rates.

Finally, larger hospitals (“Hospital Group”), as well as patients with a greater number of medical and procedural complexities, also predict greater mortality. In this line, we observe that not only individual variables but also context variables (length of stay, procedural effort and hospital group), become direct predictors of the prevalence of exitus (see Supplementary Material). Other studies have examined the greater efficiency of small hospitals in comparison to large complexes, especially when implanting pacemakers with and without associated HF [31].

Mortality is also associated with the main contextual variable readmissions, whereby a reasonable, logical connection is established between the two main dependent variables, one from each dimension (individual vs. contextual). Thus, in our model, readmission predicts greater probability of mortality, as is attested to and consistent with the previous literature, mainly the CHARM study [17], where death rates clearly increase after hospitalizations for HF even after adjusting for known mortality predictors; according to the literature, this risk increases one month after discharge and continues to rise gradually. The structural model must be supplied with a logical, plausible interpretation such that there is an obvious (but mathematically evident) association where exitus negatively predicts readmissions—for obvious reasons, but accounted for in the model from the mathematical viewpoint.

The study has found significant, direct, linear predictive relationships for the likelihood of 30-day readmission for HF. Namely, the existence of anemia, RI, diabetes and COPD are objectified as comorbidities that increase the risk of readmission, and this is well known from the prior evidence and the literature [17,23,32]. In the same sense, a direct prediction from NDX may be an expression that patients with greater diagnostic complexity and comorbidities have a greater likelihood of readmission, while those who receive greater therapeutic effort (NPR) present lower risk. The relationship between number and type of comorbidities and the risk of readmission and in-hospital death has been explored by different predictive models; they are assigned different weightings, but are a constant in the modeling of these phenomena [16,33].

It is important to stress once again the role of *contextual factors* in the risk of readmission (which in turn increases mortality). In this line of argument, the length of stay did not decree an association that would allow predicting readmissions; we understand that this can be interpreted as meaning that the short length of stay in the short-stay units does not affect readmissions and, therefore, does not detract from their efficiency [34]. The direction of prediction for the proxy variables of comorbidity (NDX) and procedural effort (NPR) is totally consistent with medical logic and biological plausibility. Thus, the more complex patients, with a greater number of diagnoses and greater disease burden, are those with the most readmissions and, secondarily, with a higher death rate; concurrently, those who received greatest therapeutical effort have lower risk of readmission.

Indirectly, the year also significantly and directly predicts readmission, probably because the group of readmitting patients is obviously more elderly (direct path) and because NDX increases over the years in a population that is increasingly more aged and complex (indirect path).

Readmission was predicted by a number of well-understood entities (anemia, RI, diabetes, COPD) as well as by patients’ level of comorbidity (NDX).

Finally, it is important to note that readmission shows some apparently paradoxical behaviors, as in a negative prediction from dyslipidemia and arrhythmias. This behavior may be related to Jencks’ bias of underreporting, well known in this type of study using clinical-administrative databases [35].

### 4.2. Second-Level Model

In the simplified model, the relationships previously exposed have been verified with greater clarity. It has been shown that dimension D1 (age, gender and COPD) positively predicts D6 (DEATH). However, there are two dimensions that negatively predict D6 (DEATH); on the one hand, D2 (PATHOLOGIES) directly predicts D5 (INTERVENTIONS) and, negatively, D6 (DEATH) and on the other, D3 (Hospital Group), which is the one that most clearly predicts D6 (DEATH) in a negative way. This result would endorse the importance of hospital groups to be a mediating variable between pathology and death through the interventions carried out, with a clear increase during the ten years analyzed. Furthermore, indirect predictions confirm this trend: although the pathologies analyzed predict death associated with cardiovascular factors, large hospitals, with greater interventions and their improvement over time (in the 10 years analyzed), have slowed this probability. Consequently, hospital contextual factors have a very relevant weight to modulate the prediction of death due to cardiovascular reasons (Supplementary Material, Model S2).

### 4.3. Limitations

The present work has several limitations that need to be clearly stated and established. First, the source of information (Minimum Basic Data Set) suffers from a series of well-known problems which, in themselves, are both a challenge and a limitation.

On the one hand, the well-known Jencks bias [35] or under-coding of diagnoses corresponding to chronic comorbidities can modify the strength and direction of the association in some cases, as occurs in hypertension and dyslipidemia. In dealing with this limitation, it is essential to review the literature and prioritize the clinical meaning of the associations so as not to obtain spurious associations. In fact, some variables that had little consistency in previous works were not considered (such as psychological disorders and previous surgeries). Although there may be a selection bias, it is precisely the information bias of the BMDS that may explain this underreporting more precisely [36]. Furthermore, there is no “operative” definition for the diagnosis of certain comorbidities, so the fact that they appear in the clinical history and have been coded by a medical specialist in documentation is the only criterion for considering them valid. Consequently, this inclusion of comorbidities based on the clinical history but not on exact definitions of the comorbidities, a limitation imposed by the characteristics of the BMDS, should be assumed as a limitation of this study.

Finally, it should be recalled that, overall, this project may be affected by the limitations inherent in the use of clinical-administrative databases.

## 5. Conclusions

We conclude then, with reasonable support from prior evidence, and from logic and the biological plausibility of how HF evolves, that it is possible to create a prediction model of mortality in this entity and incorporate contextual factors to the extent that they prove indispensable. We feel that once the theoretical models obtained from big databases take contextual variables into account, any model based exclusively on individual variables will inevitably lack elements that can explain part of the variability of this phenomenon.

The model finally obtained provides interesting information that explains the complex relationships between mortality and FH.

Death is conditioned or predicted by multiple factors, but the weight of age, sex and suffering from COPD pathology are determining factors according to our results.

Other variables are associated with lower mortality and provide interesting conclusions. Mortality is lower the fewer diagnoses patients have coded, which translates into the need to code more extensively those patients who die (usually brief and not very detailed reports of very serious patients who die shortly after admission). It is also interesting from the point of view of management that the larger hospitals report higher mortality than the rest, all mediated by the intermediate variables studied.

Direct linear predictive relationships were found between 30-day readmission and different predictors such as anemia, renal failure, COPD and diabetes. From a quantitative point of view, reference is made to the concrete values shown in the “Standardized Direct Effects” (Default model 1) tables on pages 21 and 22 of the Supplementary Material, where the level of the standardized direct effect (and indirect effect on page 23) is given. Similarly, the relationship between “Hospital Group” and diagnostic-therapeutic complexities is statistically and methodologically consistent, as shown in the Supplementary Material.

From a forward-looking viewpoint, in addition to contributing a new modeling approach with an original outlook on the study of factors associated with HF mortality, we feel that future studies and modeling of any aspect of HF should consider the importance of the context. We must incorporate more contextual variables, make use of big data and integrate multiple relational databases in order for clinical data about the individual to be contextualized in an environment of external variables, where the accuracy of estimates will undoubtedly be greater than with the exclusive use of individual variables.

We expect that the present study will constitute a point of departure toward new lines of research where management-related, contextual and environmental variables become elements that help improve the accuracy of our calculations. Strictly individual elements cannot and should not be the only elements intervening in creation of models for entities that are so complex and that form part of multiple morbid processes, as in the case of HF.

## Figures and Tables

**Table 1:** Constituent variables of the model classified along two axes, and coding of variables in this investigation.

VARIABLES CLASSIFIED ALONG TWO AXES OF ANALYSIS			
	PREDICTORS VARIABLES	MEDIATING VARIABLES	CRITERIA VARIABLES
**INDIVIDUAL VAR.**	Age	HTA	**Mortality**
	Gender	PE	
	Anemia	Arrhythmias	
	Renal Insufficiency Diabetes		
**CONTEXTUAL VAR.**	Year	Length of Stay	**Readmission**
	Hospital Group	Num- of Diagnoses	
		Num- of Procedures	
**VARIABLES STUDIED ACCORDING TO CONTEXT**			
**Patient variables**	**In-hospital context variable**		
Age (years)	Year (2008 to 2012)		
Gender (M/F) (%)	Hospital Group (administrative status, I to IV)		
Anemia (%)	Length of stay (number of days of hospital stay)		
Renal insufficiency (%)	NDX (number, continuous discrete)		
Diabetes (%)	NPR (number, continuous discrete)		
Hypertension (%)	Readmissions (Yes/No)		
Pulmonary Embolism (%)			
Arrhythmias (Yes/No)			
Dyslipidemia (Yes/No)			
COPD (%)			
Exitus (%)			

*Note:* NDX: Number of diagnoses at discharge. NPR: Number of procedures at discharge. COPD: chronic obstructive pulmonary disease.

**Table 2:** Correlation between variables (*n* = 529.606).

	1	2	3	4	5	6	7	8	9	10	11	12	13	14	15	16	17
1. Age																	
2. Gender	0.221																
3. Anemia	0.075	0.054															
4. RI	0.055	−0.065	0.108														
5. Diabetes	−0.090	−0.050	0.022		0.032												
6. Year	0.096	0.005	0.098	0.051	0.085												
7. HospGr	−0.042	−0.007	0.016	−0.013	0.018	0.019											
8. HTA	0.033	0.136	−0.030	−0.041	0.137	0.121	0.023										
9. PE	0.011	0.010	−0.003		−0.012	−0.004	0.007	−0.009									
10. Arrhy	0.070	0.038	−0.018	−0.033	−0.058	0.097	0.012	0.035	0.007								
11. Dyslip.	−0.103	0.003	−0.010	−0.010	0.139	0.151	0.031	0.143	−0.007	−0.024							
12. COPD	−0.029	−0.227	−0.033	−0.088	−0.021		0.013	−0.033	−0.005	−0.005	−0.018						
13. Stay	−0.027	0.006	0.079	0.032	0.032	−0.041	0.067	−0.024	0.016	0.020	−0.014	0.037					
14. NDX	−0.012	−0.046	0.221	0.051	0.252	0.390	0.095	0.178	0.019	0.224	0.224	0.146	0.179				
15. NPR	−0.029	−0.010	0.101	−0.013	0.051	0.202	0.006	0.052	0.023	0.082	0.073	0.028	0.198	0.369			
16. Readm	−0.009	−0.027	0.027	0.040	0.032	0.024	−0.003	−0.022	0.003	−0.012	0.004	0.025	0.002	0.043	−0.029		
17. Exitus	0.116	0.009	−0.004	0.106	−0.034	−0.004	0.010	0.079	0.062	−0.008	−0.056	−0.007	−0.107	0.003	−0.002	0.040	

Note: All values are significant at *p* < 0.001.

**Table 3:** Models of structural linear of second-level results of the variables.

Model	Degrees of Freedom	Chi-Square	*p*<	NFI	RFI	IFI	TLI	CFI	RMSEA	Hoelter
										0.05–0.01
1. 17 F	(170–121): 49	157,341.89	0.001	0.825	0.454	0.825	0.454	0.854	0.063	341–385
2. 17 F	(170–138): 32	71,268.011	0.001	0.921	0.621	0.921	0.621	0.921	0.052	524–607
3. 17 F	(170–151): 19	1926.507	0.001	0.946	0.941	0.946	0.941	0.946	0.050	497–541

*Note.* Models 1 and 2 (complex with 17 factors); model 3 (simplified with 6 dimensions). NFI: normed fit index; RFI: relative fit index; IFI: incremental fit index; TLI: Tucker–Lewis index; CFI: corporative fit index; RMSEA: root-mean-square error of approximation.

**Table 4:** Standardized DIRECT effects (default model).

	D1	D2	D3	D4	D5	D6
D1. PATIENS						
D2. PATHOLOG.	0.136		0.582			
D3. TYPE HOSPI	0.060					
D4. YEAR			0.921			
D5. INTERVENT	−0.122	0.131		0.870		
D6. DEATH	0.103	−0.147	−0.887		0.713	
Gender	0.974					
Age	0.223					
COPD	−0.282					
Diabetes		0.391				
Dyslipemia		0.372				
HTA		0.352				
EP			0.027			
HOSPGR			0.104			
Year				0.358		
ARR				0.234		
IR						0.599
NDX					0.769	
STAY					0.157	
NPR					0.319	
Anemia					0.195	
READ						0.087
Exitus						0.170

*Note:* D1 = PRESAGE FACTORS OF PATIENS; D2 = PROCESS FACTORS OF PATTIENTS: ASSOCIATED PATHOLOGIES; D3 = PRESAGE FACTOR OF CONTEXT: TYPE OF HOSPITAL;D4 = PROCESS OF CONTEXT: YEARS; D5 = PROCESS OF CONTEXT: INTERVENTIONS; D6 = PRODUCT OF PATIENTS: DEATH; HTA = arterial hypertension; ARR = arrhythmias; COPD = chronic obstructive pulmonary disease; YEAR = year; HOSPGR = hospital group; EP = pulmonary thromboembolism; STAY = length of stay; NDX = number of diagnoses; NPR = number of procedures; READ = readmission. EXITUS = death.

**Table 5:** Standardized Indirect Effects (Default model).

	D1	D2	D3	D4	D5	D6
D1. PATIENS						
D2. PATHOLOG.	0.035					
D3. TYPE HOSPI						
D4. YEAR	−0.055					
D5. INTERVENT	0.070		0.877			
D6. DEATH	−0.208	0.224	−0.417	−0.491		
Gender						
Age						
COPD						
Diabetes	0.067		0.228			
Dyslipemia	0.064		0.217			
HTA	0.060		0.205			
EP	0.002					
Hospgrup	0.006					
Year	0.020		0.330			
ARR	0.013		0.216			
IR	−0.063	0.046	−0.101	0.892	0.825	
NDX	−0.055	0.140	0.938	0.930		
Stance	−0.008	0.021	0.138	0.137		
NPR	−0.016	0.042	0.280	0.278		
Anemia	−0.010	0.025	0.171	0.170		
Reentry	−0.009	0.007	−0.015	0.130	0.150	
Exitus	−0.018	0.013	−0.029	0.253	0.290	

*Note:* D1 = PRESAGE FACTORS OF PATIENS; D2 = PROCESS FACTORS OF PATTIENTS: ASSOCIATED PATHOLOGIES; D3 = PRESAGE FACTOR OF CONTEXT: TYPE OF HOSPITAL; D4 = PROCESS OF CONTEXT: YEARS; D5 = PROCESS OF CONTEXT: INTERVENTIONS; D6 = PRODUCT OF PATIENTS: DEATH; AGE = age; GEND = gender; ANEM = anemia; DIAB = diabetes; HTA = arterial hypertension; ARR = arrhythmias; COPD = chronic obstructive pulmonary disease; YEAR = year; HOSPGR = hospital group; TEP = pulmonary thromboembolism; STAY = length of stay; NDX = number of diagnoses; NPR = number of procedures; READ = readmission. EXITUS = death.

## Data Availability

The datasets generated and analysed during the current study are not publicly available, in accordance with Law 679/2016 of the European Parliament, but are available from the corresponding author on reasonable request.

## Supplementary Materials

The following supporting information can be downloaded at: https://www.mdpi.com/article/10.3390/jpm13060995/s1, Table S1: COMPARISON ACCORDING TO MORTALITY GROUPS; Model S1: SIMPLIFIED FIRST-LEVEL MODEL FOR APPROXIMATING HOSPITAL MORTALITY; Model S2: ADVANCED SECOND-TIER MODEL FOR HOSPITAL MORTALITY.
