# Supplementary material for: Structural Model of Biomedical and Contextual Factors Predicting In-Hospital Mortality due to Heart Failure
Source: J Pers Med. 2023 Jun 13;13(6):995. doi: 10.3390/jpm13060995 (PMC10301776; doi:10.3390/jpm13060995)
Supplement: Supplementary file 1 [file jpm-13-00995-s001.zip › jpm-2408216-supplementary.pdf]

COMPLEMENTARY MATERIAL

Table S1. COMPARISON ACCORDING TO MORTALITY GROUPS

Basal distribution of the studied variables according to mortality groups			
	Living patients	Exitus	p-value
Arterial hypertension	12.4	8.2	<0.001
Ischemic heart disease	10.3	9.4	<0.001
COPD	10.3	9.5	<0.001
Valvulopathies	10.8	8.7	<0.001
Atrial Fibrillation	11.1	9.3	<0.001
Ischemic Stroke	9.9	15	<0.001
Hemorrhagic stroke	10.2	33.2	<0.001
Kidney Failure	14.4	8.2	<0.001
Anemia	10.3	9.8	<0.001
Pulmonary thromboembolism	10.1	31.6	<0.001
Atrial Fibrillation	11.1	9.3	<0.001
Dyslipemia	11.3	7	<0.001
Obesity	6.5	10.7	<0.001
Diabetes	11.1	8.6	<0.001
Distribution of variables according to Living patients vs Exitus			
Sexo Mujer	89.7	10.3	
Sexo Hombre	90	10	<0.001
Ingreso Programado	90.5	9.5	
Ingreso Urgente	89.8	10.2	<0.001
Readmission Yes	86.2	13.8	

Readmissin No	90.6	9.4	<0.001
Quantitative variables studied [Mean (Standard Deviation)			
	Alive	Exitus	p-value
Age	78.68(10.61)	82.05(10.37)	<0.001
Length of stay	7.39(4.38)	6.36(5.38)	<0.001
NDX	9.30(2.88)	9.43(3.04)	<0.001
NPR	2.60(2.59)	2.66(2.91)	<0.001

NDX: Number of diagnoses at discharge. NPR: Number of procedures at discharge

Model S1. SIMPLIFIED FIRST-LEVEL MODEL FOR APPROXIMATING HOSPITAL MORTALITY .

Notes for Model (Default model)

Computation of degrees of freedom (Default model)

Number of distinct sample moments:	170
Number of distinct parameters to be estimated:	138
Degrees of freedom (170 - 138):	32

Result (Default model)

Minimum was achieved
Chi-square = 71268.011
Degrees of freedom = 32
Probability level = 0.000

Estimates (Group number 1 - Default model)

Scalar Estimates (Group number 1 - Default model)

Maximum Likelihood Estimates

Regression Weights: (Group number 1 - Default model)

			Estimate	S.E.	C.R.	P	Label
GENDER	<---	AGE	0.011	0.000	203.339	***	
ANEMIA	<---	GENDER	0.037	0.001	48.671	***	
HOSPGR	<---	AGE	-0.004	0.000	-39.458	***	
DIABETES	<---	GENDER	0.046	0.001	44.037	***	
RENALINS	<---	AGE	0.002	0.000	58.045	***	
RENALINS	<---	GENDER	-0.060	0.001	-75.331	***	
RENALINS	<---	ANEMIA	0.112	0.001	98.108	***	
DIABETES	<---	ANEMIA	0.026	0.002	17.077	***	
HOSPGR	<---	YEAR	0.007	0.000	20.737	***	
HTA	<---	DIABETES	0.154	0.001	130.098	***	
HTA	<---	HOSPGR	0.011	0.001	20.042	***	
HTA	<---	AGE	0.003	0.000	44.957	***	
HTA	<---	RENALINS	-0.069	0.002	-44.029	***	
ARRYHT	<---	GENDER	0.047	0.001	40.582	***	
DIAGN	<---	YEAR	0.335	0.001	334.157	***	
EPOC	<---	RENALINS	-0.076	0.005	-16.175	***	
EPOC	<---	ANEMIA	-0.129	0.009	-14.342	***	
DYSLIPEM	<---	ANEMIA	-0.079	0.001	-57.036	***	
DIAGN	<---	HOSPGR	0.186	0.003	65.371	***	
PRODEC	<---	YEAR	0.065	0.001	80.248	***	
DYSLIPEM	<---	GENDER	0.025	0.001	32.130	***	
EPOC	<---	AGE	0.002	0.000	14.286	***	
EPOC	<---	GENDER	-0.230	0.001	-179.508	***	
ARRYHT	<---	ANEMIA	-0.157	0.005	-34.782	***	
TEP	<---	HTA	-0.001	0.000	-5.989	***	
ARRYHT	<---	HTA	-0.018	0.001	-14.744	***	
DYSLIPEM	<---	HTA	0.043	0.001	55.364	***	
TEP	<---	DIABETES	-0.002	0.000	-10.478	***	
DYSLIPEM	<---	DIABETES	0.016	0.001	18.043	***	
EPOC	<---	DIABETES	-0.030	0.004	-7.220	***	
ARRYHT	<---	DIABETES	-0.150	0.002	-67.877	***	
EPOC	<---	HOSPGR	-0.022	0.002	-10.168	***	
ARRYHT	<---	HOSPGR	-0.014	0.001	-11.837	***	
TEP	<---	HOSPGR	0.000	0.000	6.106	***	
STANCE	<---	DIABETES	0.168	0.035	4.867	***	
STANCE	<---	ANEMIA	0.705	0.032	22.270	***	
STANCE	<---	AGE	0.010	0.001	6.860	***	
PRODEC	<---	AGE	-0.008	0.000	-30.582	***	
DYSLIPEM	<---	AGE	-0.003	0.000	-84.078	***	
ARRYHT	<---	AGE	0.003	0.000	49.261	***	
DIAGN	<---	ANEMIA	1.335	0.009	154.164	***	
DIAGN	<---	RENALINS	0.981	0.008	120.489	***	
PRODEC	<---	RENALINS	-0.307	0.008	-40.107	***	
PRODEC	<---	HOSPGR	-0.093	0.003	-37.022	***	
ARRYHT	<---	RENALINS	-0.121	0.003	-43.988	***	
DYSLIPEM	<---	RENALINS	-0.056	0.001	-48.803	***	
STANCE	<---	HOSPGR	0.413	0.009	44.151	***	
STANCE	<---	GENDER	-2.868	0.235	-12.200	***	
DIAGN	<---	GENDER	-0.263	0.007	-37.421	***	

			Estimate	S.E.	C.R.	P	Label
PRODEC	<---	GENDER	-0.035	0.009	-3.793	***	
DIAGN	<---	DIABETES	1.270	0.006	199.618	***	
PRODEC	<---	DIABETES	-0.233	0.005	-42.385	***	
DIAGN	<---	HTA	0.856	0.006	144.433	***	
PRODEC	<---	HTA	-0.034	0.005	-7.048	***	
REENTRY	<---	DIAGN	0.006	0.000	40.165	***	
REENTRY	<---	PRODEC	-0.007	0.000	-42.198	***	
REENTRY	<---	ARRYHT	-0.010	0.001	-14.367	***	
REENTRY	<---	DYSLIPEM	-0.004	0.001	-3.715	***	
REENTRY	<---	EPOC	0.014	0.001	15.938	***	
REENTRY	<---	HTA	-0.018	0.001	-25.971	***	
REENTRY	<---	TEP	0.017	0.006	3.004	.003	
REENTRY	<---	RENALINS	0.024	0.001	23.978	***	
REENTRY	<---	ANEMIA	0.014	0.001	13.447	***	
REENTRY	<---	HOSPGR	-0.002	0.000	-7.097	***	
REENTRY	<---	DIABETES	0.016	0.001	20.344	***	
EXITUS	<---	DYSLIPEM	-0.044	0.001	-42.146	***	
EXITUS	<---	EPOC	-0.011	0.001	-12.903	***	
EXITUS	<---	REENTRY	0.037	0.001	34.814	***	
EXITUS	<---	ARRYHT	-0.016	0.001	-22.820	***	
EXITUS	<---	HTA	-0.053	0.001	-78.604	***	
EXITUS	<---	TEP	0.298	0.005	54.969	***	
EXITUS	<---	AGE	0.004	0.000	107.609	***	
EXITUS	<---	YEAR	-0.003	0.000	-24.113	***	
EXITUS	<---	ANEMIA	-0.020	0.001	-19.729	***	
EXITUS	<---	PRODEC	0.003	0.000	16.877	***	
EXITUS	<---	STANCE	-0.008	0.000	-108.459	***	
EXITUS	<---	HOSPGR	0.007	0.000	19.803	***	
EXITUS	<---	DIAGN	0.007	0.000	46.844	***	
STANCE	<---	ARRYHT	-3.080	0.261	-11.793	***	
DIAGN	<---	STANCE	0.159	0.002	87.362	***	
PRODEC	<---	DIAGN	0.273	0.001	238.841	***	
EPOC	<---	DYSLIPEM	-0.070	0.011	-6.162	***	
PRODEC	<---	STANCE	0.088	0.003	30.272	***	
EPOC	<---	STANCE	0.073	0.006	12.908	***	
EPOC	<---	DIAGN	0.013	0.001	8.464	***	
DYSLIPEM	<---	DIAGN	0.046	0.000	133.214	***	
ARRYHT	<---	DIAGN	0.060	0.001	66.632	***	
EPOC	<---	PRODEC	0.016	0.002	6.838	***	
DYSLIPEM	<---	TEP	-0.065	0.006	-11.087	***	
EPOC	<---	TEP	-0.071	0.014	-5.080	***	
STANCE	<---	EPOC	-13.601	1.030	-13.208	***	
DIAGN	<---	TEP	1.037	0.046	22.383	***	
PRODEC	<---	TEP	0.534	0.038	13.981	***	
DIAGN	<---	ARRYHT	-0.122	.015	-8.156	***	
STANCE	<---	DYSLIPEM	-1.242	.188	-6.613	***	
DIAGN	<---	DYSLIPEM	-0.972	.023	-43.057	***	
PRODEC	<---	DYSLIPEM	-0.151	.008	-18.986	***	
DIAGN	<---	EPOC	0.928	.019	49.950	***	

			Estimate	S.E.	C.R.	P	Label
PRODEC	<---	EPOC	-0.425	.034	-12.432	***	
DYSLIPEM	<---	ARRYHT	-0.051	.002	-30.471	***	
EPOC	<---	ARRYHT	0.130	.011	11.526	***	
DYSLIPEM	<---	STANCE	-0.006	.001	-8.887	***	
ARRYHT	<---	STANCE	0.020	.003	7.606	***	
TEP	<---	STANCE	0.000	.000	8.765	***	

Standardized Regression Weights: (Group number 1 - Default model)

			Estimate
GENDER	<---	AGE	0.221
ANEMIA	<---	GENDER	0.054
HOSPGR	<---	AGE	-0.044
DIABETES	<---	GENDER	0.049
RENALINS	<---	AGE	0.066
RENALINS	<---	GENDER	-0.085
RENALINS	<---	ANEMIA	0.108
DIABETES	<---	ANEMIA	0.019
HOSPGR	<---	YEAR	0.023
HTA	<---	DIABETES	0.143
HTA	<---	HOSPGR	0.022
HTA	<---	AGE	0.049
HTA	<---	RENALINS	-0.048
ARRYHT	<---	GENDER	0.047
DIAGN	<---	YEAR	0.369
EPOC	<---	RENALINS	-0.068
EPOC	<---	ANEMIA	-0.111
DYSLIPEM	<---	ANEMIA	-0.080
DIAGN	<---	HOSPGR	0.066
PRODEC	<---	YEAR	0.090
DYSLIPEM	<---	GENDER	0.037
EPOC	<---	AGE	0.053
EPOC	<---	GENDER	-0.292
ARRYHT	<---	ANEMIA	-0.106
TEP	<---	HTA	-0.007
ARRYHT	<---	HTA	-0.018
DYSLIPEM	<---	HTA	0.064
TEP	<---	DIABETES	-0.012
DYSLIPEM	<---	DIABETES	0.023
EPOC	<---	DIABETES	-0.036
ARRYHT	<---	DIABETES	-0.140
EPOC	<---	HOSPGR	-0.055
ARRYHT	<---	HOSPGR	-0.027
TEP	<---	HOSPGR	0.007
STANCE	<---	DIABETES	0.016
STANCE	<---	ANEMIA	0.049
STANCE	<---	AGE	0.021
PRODEC	<---	AGE	-0.034

			Estimate
DYSLIPEM	<---	AGE	-0.094
ARRYHT	<---	AGE	0.068
DIAGN	<---	ANEMIA	0.158
DIAGN	<---	RENALINS	0.121
PRODEC	<---	RENALINS	-0.047
PRODEC	<---	HOSPGR	-0.041
ARRYHT	<---	RENALINS	-0.085
DYSLIPEM	<---	RENALINS	-0.058
STANCE	<---	HOSPGR	0.085
STANCE	<---	GENDER	-0.293
DIAGN	<---	GENDER	-0.046
PRODEC	<---	GENDER	-0.008
DIAGN	<---	DIABETES	0.207
PRODEC	<---	DIABETES	-0.047
DIAGN	<---	HTA	0.150
PRODEC	<---	HTA	-0.007
REENTRY	<---	DIAGN	0.055
REENTRY	<---	PRODEC	-0.050
REENTRY	<---	ARRYHT	-0.017
REENTRY	<---	DYSLIPEM	-0.004
REENTRY	<---	EPOC	0.018
REENTRY	<---	HTA	-0.030
REENTRY	<---	TEP	0.003
REENTRY	<---	RENALINS	0.027
REENTRY	<---	ANEMIA	0.015
REENTRY	<---	HOSPGR	-0.008
REENTRY	<---	DIABETES	0.024
EXITUS	<---	DYSLIPEM	-0.048
EXITUS	<---	EPOC	-0.014
EXITUS	<---	REENTRY	0.038
EXITUS	<---	ARRYHT	-0.026
EXITUS	<---	HTA	-0.087
EXITUS	<---	TEP	0.060
EXITUS	<---	AGE	0.118
EXITUS	<---	YEAR	-0.028
EXITUS	<---	ANEMIA	-0.022
EXITUS	<---	PRODEC	0.020
EXITUS	<---	STANCE	-0.123
EXITUS	<---	HOSPGR	0.022
EXITUS	<---	DIAGN	0.064
STANCE	<---	ARRYHT	-0.317
DIAGN	<---	STANCE	0.272
PRODEC	<---	DIAGN	0.341
EPOC	<---	DYSLIPEM	-0.060
PRODEC	<---	STANCE	0.187
EPOC	<---	STANCE	0.904
EPOC	<---	DIAGN	0.092
DYSLIPEM	<---	DIAGN	0.390
ARRYHT	<---	DIAGN	0.340

			Estimate
EPOC	<---	PRODEC	0.093
DYSLIPEM	<---	TEP	-0.012
EPOC	<---	TEP	-0.011
STANCE	<---	EPOC	-1.097
DIAGN	<---	TEP	0.022
PRODEC	<---	TEP	0.014
DIAGN	<---	ARRYHT	-0.021
STANCE	<---	DYSLIPEM	-0.086
DIAGN	<---	DYSLIPEM	-0.115
PRODEC	<---	DYSLIPEM	-0.022
DIAGN	<---	EPOC	0.128
PRODEC	<---	EPOC	-0.073
DYSLIPEM	<---	ARRYHT	-0.076
EPOC	<---	ARRYHT	0.166
DYSLIPEM	<---	STANCE	-0.084
ARRYHT	<---	STANCE	0.196
TEP	<---	STANCE	0.014

Intercepts: (Group number 1 - Default model)

	Estimate	S.E.	C.R.	P	Label
AGE	77.140	0.011	7069.692	***	
GENDER	0.700	0.004	163.902	***	
ANEMIA	0.074	0.001	59.528	***	
YEAR	2002.508	0.003	573344.514	***	
RENALINS	0.042	0.003	13.461	***	
HOSPGR	-11.926	0.711	-16.778	***	
DIABETES	0.240	0.002	140.814	***	
HTA	0.216	0.005	46.740	***	
ARRYHT	-0.255	0.026	-9.880	***	
DYSLIPEM	0.099	0.007	13.490	***	
EPOC	-0.284	0.054	-5.230	***	
STANCE	14.919	0.426	35.061	***	
DIAGN	-666.327	2.006	-332.164	***	
PRODEC	-129.745	1.623	-79.961	***	
TEP	0.002	0.000	8.927	***	
REENTRY	0.087	0.001	72.197	***	
EXITUS	5.376	0.229	23.497	***	

Variances: (Group number 1 - Default model)

	Estimate	S.E.	C.R.	P	Label
e1	96.225	0.151	635.700	***	
e2	0.234	0.000	635.676	***	
e3	0.113	0.000	635.699	***	
e5	9.859	0.016	635.700	***	

	Estimate	S.E.	C.R.	P	Label
e4	0.120	0.000	635.699	***	
e15	1.004	0.002	635.642	***	
e16	0.215	0.000	635.700	***	
e18	0.244	0.000	635.700	***	
e8	0.104	0.000	582.089	***	
e9	0.267	0.020	13.600	***	
e10	54.237	3.850	14.087	***	
e11	6.273	0.020	315.319	***	
e12	4.363	0.008	523.235	***	
e7	0.242	0.003	81.338	***	
e19	0.004	0.000	635.695	***	
e14	0.095	0.000	635.700	***	
e13	0.089	0.000	635.700	***	

Matrices (Group number 1 - Default model)

Implied Covariances (Group number 1 - Default model)

	AGE	GENDER	YEAR	ANEMIA	DIABETES	HOSPGR	RENALINS	HTA	TEP	STANCE	ARRYHT	PRODEC	DIAGN	EPOC	DYSLIPEM	REENTRY	EXITUS
AGE	96.225																
GENDER	1.074	0.246															
YEAR	0.000	0.000	9.859														
ANEMIA	0.039	0.009	0.000	0.114													
DIABETES	0.050	0.012	0.000	0.003	0.216												
HOSPGR	-0.431	-0.005	0.073	0.000	0.000	1.006											
RENALINS	0.165	-0.011	0.000	0.012	0.000	-0.001	0.123										
HTA	0.234	0.005	0.001	0.000	0.033	0.010	-0.008	0.250									
TEP	-0.001	0.000	0.000	0.000	0.000	0.000	0.000	0.000	0.004								
STANCE	-1.425	0.019	-0.806	0.140	0.069	0.318	0.077	-0.032	0.005	23.573							
ARRYHT	0.327	0.008	0.164	-0.005	-0.014	0.007	-0.006	0.005	0.000	0.046	0.250						
PRODEC	-0.863	-0.016	1.370	0.060	0.033	0.011	0.010	0.038	0.003	2.187	0.094	5.199					
DIAGN	0.033	-0.092	3.046	0.186	0.295	0.264	0.147	0.221	0.004	2.498	0.322	2.348	8.115				
EPOC	-0.107	-0.054	0.012	-0.005	-0.003	0.005	-0.001	-0.001	0.000	0.068	-0.001	0.027	0.174	0.153			
DYSLIPEM	-0.292	-0.002	0.136	-0.002	0.019	0.012	-0.002	0.021	0.000	-0.024	-0.004	0.051	0.229	-0.002	0.113		
REENTRY	0.004	-0.001	0.007	0.003	0.004	-0.001	0.004	-0.003	0.000	0.005	-0.002	-0.021	0.037	0.003	0.000	0.096	
EXITUS	0.355	0.003	-0.004	-0.001	-0.001	0.003	0.002	-0.012	0.001	-0.160	-0.001	-0.001	0.006	-0.001	-0.005	0.004	0.093

Implied Correlations (Group number 1 - Default model)

	AGE	GENDER	YEAR	ANEMIA	DIABETES	HOSPGR	RENALINS	HTA	TEP	STANCE	ARRYHT	PRODEC	DIAGN	EPOC	DYSLIPEM	REENTRY	EXITUS
AGE	1.000																
GENDER	0.221	1.000															
YEAR	0.000	0.000	1.000														
ANEMIA	0.012	0.054	0.000	1.000													
DIABETES	0.011	0.050	0.000	0.022	1.000												
HOSPGR	-0.044	-0.010	0.023	-0.001	0.000	1.000											
RENALINS	0.048	-0.065	0.000	0.104	-0.001	-0.002	1.000										
HTA	0.048	0.021	0.001	-0.001	0.143	0.020	-0.046	1.000									
TEP	-0.001	-0.001	-0.001	0.001	-0.012	0.008	0.001	-0.008	1.000								
STANCE	-0.030	0.008	-0.053	0.085	0.031	0.065	0.046	-0.013	0.016	1.000							
ARRYHT	0.067	0.034	0.105	-0.032	-0.060	0.013	-0.035	0.020	0.009	0.019	1.000						
PRODEC	-0.039	-0.015	0.191	0.078	0.031	0.005	0.012	0.033	0.025	0.198	0.083	1.000					
DIAGN	0.001	-0.065	0.340	0.193	0.223	0.092	0.147	0.155	0.022	0.181	0.226	0.362	1.000				
EPOC	-0.028	-0.277	0.010	-0.037	-0.018	0.013	-0.007	-0.005	-0.003	0.036	-0.004	0.030	0.156	1.000			
DYSLIPEM	-0.089	-0.011	0.129	-0.014	0.120	0.035	-0.020	0.126	-0.006	-0.014	-0.025	0.067	0.239	-0.013	1.000		
REENTRY	0.001	-0.009	0.007	0.025	0.030	-0.004	0.038	-0.022	0.003	0.004	-0.012	-0.030	0.042	0.024	0.004	1.000	
EXITUS	0.119	0.021	-0.005	-0.014	-0.004	0.010	0.015	-0.077	0.061	-0.108	-0.007	-0.001	0.007	-0.009	-0.053	0.041	1.000

Implied Means (Group number 1 - Default model)

	AGE	GENDER	YEAR	ANEMIA	DIABETES	HOSPGR	RENALINS	HTA	TEP	STANCE	ARRYHT	PRODEC	DIAGN	EPOC	DYSLIPEM	REENTRY	EXITUS
	77.140	1.561	2002.508	0.131	0.315	2.468	0.143	0.476	0.004	8.053	0.522	1.854	6.716	0.189	0.131	0.107	0.103

Standardized Total Effects (Group number 1 - Default model)

	AGE	GENDER	YEAR	ANEMIA	DIABETES	HOSPGR	RENALINS	HTA	TEP	STANCE	ARRYHT	PRODEC	DIAGN	EPOC	DYSLIPEM	REENTRY
GENDER	0.221	0.000	0.000	0.000	0.000	0.000	0.000	0.000	0.000	0.000	0.000	0.000	0.000	0.000	0.000	0.000
ANEMIA	0.012	0.054	0.000	0.000	0.000	0.000	0.000	0.000	0.000	0.000	0.000	0.000	0.000	0.000	0.000	0.000
DIABETES	0.011	0.050	0.000	0.019	0.000	0.000	0.000	0.000	0.000	0.000	0.000	0.000	0.000	0.000	0.000	0.000
HOSPGR	-0.044	0.000	0.023	0.000	0.000	0.000	0.000	0.000	0.000	0.000	0.000	0.000	0.000	0.000	0.000	0.000
RENALINS	0.048	-0.079	0.000	0.108	0.000	0.000	0.000	0.000	0.000	0.000	0.000	0.000	0.000	0.000	0.000	0.000
HTA	0.048	0.011	0.001	-0.003	0.143	0.022	-0.048	0.000	0.000	0.000	0.000	0.000	0.000	0.000	0.000	0.000
TEP	-0.001	0.000	-0.001	0.001	-0.012	0.008	0.001	-0.007	0.000	0.006	-0.003	-0.001	-0.002	-0.007	0.000	0.000
STANCE	-0.030	0.015	-0.053	0.085	0.029	0.065	0.040	-0.016	0.002	-0.550	-0.225	-0.047	-0.136	-0.508	0.008	0.000
ARRYHT	0.067	0.020	0.105	-0.034	-0.061	0.014	-0.034	0.024	0.008	0.148	-0.071	-0.012	0.286	-0.125	-0.038	0.000
PRODEC	-0.039	-0.006	0.191	0.079	0.031	-0.001	0.005	0.032	0.023	0.111	-0.063	-0.015	0.279	-0.158	-0.054	0.000
DIAGN	0.001	-0.068	0.340	0.197	0.223	0.085	0.123	0.132	0.023	0.175	-0.079	-0.007	-0.081	-0.075	-0.116	0.000
EPOC	-0.028	-0.285	0.010	-0.022	-0.003	0.012	-0.025	-0.002	-0.004	0.457	-0.057	0.046	0.014	-0.503	-0.072	0.000
DYSLIPEM	-0.089	0.009	0.129	-0.014	0.121	0.028	-0.014	0.115	-0.004	0.019	-0.082	0.002	0.348	0.023	-0.043	0.000
REENTRY	0.001	-0.009	0.007	0.026	0.031	-0.004	0.035	-0.025	0.003	0.010	-0.017	-0.049	0.031	0.015	-0.008	0.000
EXITUS	0.119	-0.006	-0.005	-0.015	-0.005	0.016	0.011	-0.084	0.062	-0.052	0.001	0.023	0.058	0.050	-0.054	0.038

Standardized Direct Effects (Group number 1 - Default model)

	AGE	GENDER	YEAR	ANEMIA	DIABETES	HOSPGR	RENALINS	HTA	TEP	STANCE	ARRYHT	PRODEC	DIAGN	EPOC	DYSLIPEM	REENTRY
GENDER	0.221	0.000	0.000	0.000	0.000	0.000	0.000	0.000	0.000	0.000	0.000	0.000	0.000	0.000	0.000	0.000
ANEMIA	0.000	0.054	0.000	0.000	0.000	0.000	0.000	0.000	0.000	0.000	0.000	0.000	0.000	0.000	0.000	0.000
DIABETES	0.000	0.049	0.000	0.019	0.000	0.000	0.000	0.000	0.000	0.000	0.000	0.000	0.000	0.000	0.000	0.000
HOSPGR	-0.044	0.000	0.023	0.000	0.000	0.000	0.000	0.000	0.000	0.000	0.000	0.000	0.000	0.000	0.000	0.000
RENALINS	0.066	-0.085	0.000	0.108	0.000	0.000	0.000	0.000	0.000	0.000	0.000	0.000	0.000	0.000	0.000	0.000
HTA	0.049	0.000	0.000	0.000	0.143	0.022	-0.048	0.000	0.000	0.000	0.000	0.000	0.000	0.000	0.000	0.000
TEP	0.000	0.000	0.000	0.000	-0.012	0.007	0.000	-0.007	0.000	0.014	0.000	0.000	0.000	0.000	0.000	0.000
STANCE	0.021	-0.293	0.000	0.049	0.016	0.085	0.000	0.000	0.000	0.000	-0.317	0.000	0.000	-1.097	-0.086	0.000
ARRYHT	0.068	0.047	0.000	-0.106	-0.140	-0.027	-0.085	-0.018	0.000	0.196	0.000	0.000	0.340	0.000	0.000	0.000
PRODEC	-0.034	-0.008	0.090	0.000	-0.047	-0.041	-0.047	-0.007	0.014	0.187	0.000	0.000	0.341	-0.073	-0.022	0.000
DIAGN	0.000	-0.046	0.369	0.158	0.207	0.066	0.121	0.150	0.022	0.272	-0.021	0.000	0.000	0.128	-0.115	0.000
EPOC	0.053	-0.292	0.000	-0.111	-0.036	-0.055	-0.068	0.000	-0.011	0.904	0.166	0.093	0.092	0.000	-0.060	0.000
DYSLIPEM	-0.094	0.037	0.000	-0.080	0.023	0.000	-0.058	0.064	-0.012	-0.084	-0.076	0.000	0.390	0.000	0.000	0.000
REENTRY	0.000	0.000	0.000	0.015	0.024	-0.008	0.027	-0.030	0.003	0.000	-0.017	-0.050	0.055	0.018	-0.004	0.000
EXITUS	0.118	0.000	-0.028	-0.022	0.000	0.022	0.000	-0.087	0.060	-0.123	-0.026	0.020	0.064	-0.014	-0.048	0.038

Standardized Indirect Effects (Group number 1 - Default model)

	AGE	GENDER	YEAR	ANEMIA	DIABETES	HOSPGR	RENALINS	HTA	TEP	STANCE	ARRYHT	PRODEC	DIAGN	EPOC	DYSLIPEM	REENTRY
GENDER	0.000	0.000	0.000	0.000	0.000	0.000	0.000	0.000	0.000	0.000	0.000	0.000	0.000	0.000	0.000	0.000
ANEMIA	0.012	0.000	0.000	0.000	0.000	0.000	0.000	0.000	0.000	0.000	0.000	0.000	0.000	0.000	0.000	0.000
DIABETES	0.011	0.001	0.000	0.000	0.000	0.000	0.000	0.000	0.000	0.000	0.000	0.000	0.000	0.000	0.000	0.000
HOSPGR	0.000	0.000	0.000	0.000	0.000	0.000	0.000	0.000	0.000	0.000	0.000	0.000	0.000	0.000	0.000	0.000
RENALINS	-0.017	0.006	0.000	0.000	0.000	0.000	0.000	0.000	0.000	0.000	0.000	0.000	0.000	0.000	0.000	0.000
HTA	-0.002	0.011	0.001	-0.003	0.000	0.000	0.000	0.000	0.000	0.000	0.000	0.000	0.000	0.000	0.000	0.000
TEP	-0.001	0.000	-0.001	0.001	-0.001	0.001	0.001	0.000	0.000	-0.008	-0.003	-0.001	-0.002	-0.007	0.000	0.000
STANCE	-0.051	0.308	-0.053	0.036	0.013	-0.020	0.040	-0.016	0.002	-0.550	0.092	-0.047	-0.136	0.589	0.094	0.000
ARRYHT	-0.002	-0.026	0.105	0.072	0.079	0.041	0.050	0.042	0.008	-0.048	-0.071	-0.012	-0.054	-0.125	-0.038	0.000
PRODEC	-0.004	0.001	0.102	0.079	0.078	0.040	0.052	0.040	0.008	-0.077	-0.063	-0.015	-0.062	-0.085	-0.032	0.000
DIAGN	0.001	-0.023	-0.029	0.039	0.016	0.019	0.003	-0.018	0.000	-0.096	-0.057	-0.007	-0.081	-0.202	-0.001	0.000
EPOC	-0.081	0.007	0.010	0.089	0.032	0.067	0.043	-0.002	0.007	-0.447	-0.223	-0.047	-0.078	-0.503	-0.012	0.000
DYSLIPEM	0.006	-0.027	0.129	0.066	0.098	0.028	0.044	0.051	0.008	0.103	-0.006	0.002	-0.042	0.023	-0.043	0.000
REENTRY	0.001	-0.009	0.007	0.011	0.007	0.004	0.008	0.005	0.000	0.010	-0.001	0.001	-0.025	-0.003	-0.004	0.000
EXITUS	0.001	-0.006	0.024	0.007	-0.005	-0.006	0.011	0.004	0.002	0.071	0.027	0.003	-0.006	0.064	-0.006	0.000

Model Fit Summary

CMIN

Model	NPAR	CMIN	DF	P	CMIN/DF
Default model	138	71268.011	32	0.000	2227.125
Saturated model	170	0.000	0		
Independence model	17	899414.627	153	0.000	5878.527

Baseline Comparisons

Model	NFI Delta1	RFI rho1	IFI Delta2	TLI rho2	CFI
Default model	0.921	0.911	0.921	0.911	0.921
Saturated model	1.000		1.000		1.000
Independence model	0.000	0.000	0.000	0.000	0.000

Parsimony-Adjusted Measures

Model	PRATIO	PNFI	PCFI
Default model	0.209	0.193	0.193
Saturated model	0.000	0.000	0.000
Independence model	1.000	0.000	0.000

NCP

Model	NCP	LO 90	HI 90
Default model	71236.011	70361.027	72117.269
Saturated model	0.000	0.000	0.000
Independence model	899261.627	896145.022	902384.505

FMIN

Model	FMIN	F0	LO 90	HI 90
Default model	0.088	0.088	0.087	0.089
Saturated model	0.000	0.000	0.000	0.000
Independence model	1.113	1.113	1.109	1.116

RMSEA

Model	RMSEA	LO 90	HI 90	PCLOSE
Default model	0.052	0.052	0.053	0.000

Model	RMSEA	LO 90	HI 90	PCLOSE
Independence model	0.085	0.085	0.085	0.000

AIC

Model	AIC	BCC	BIC	CAIC
Default model	71544.011	71544.017		
Saturated model	340.000	340.008		
Independence model	899448.627	899448.627		

ECVI

Model	ECVI	LO 90	HI 90	MECVI
Default model	0.089	0.087	0.090	0.089
Saturated model	0.000	0.000	0.000	0.000
Independence model	1.113	1.109	1.117	1.113

HOELTER

Model	HOELTER 0.05	HOELTER 0.01
Default model	524	607
Independence model	165	177

OLD FIRST LEVEL MODEL

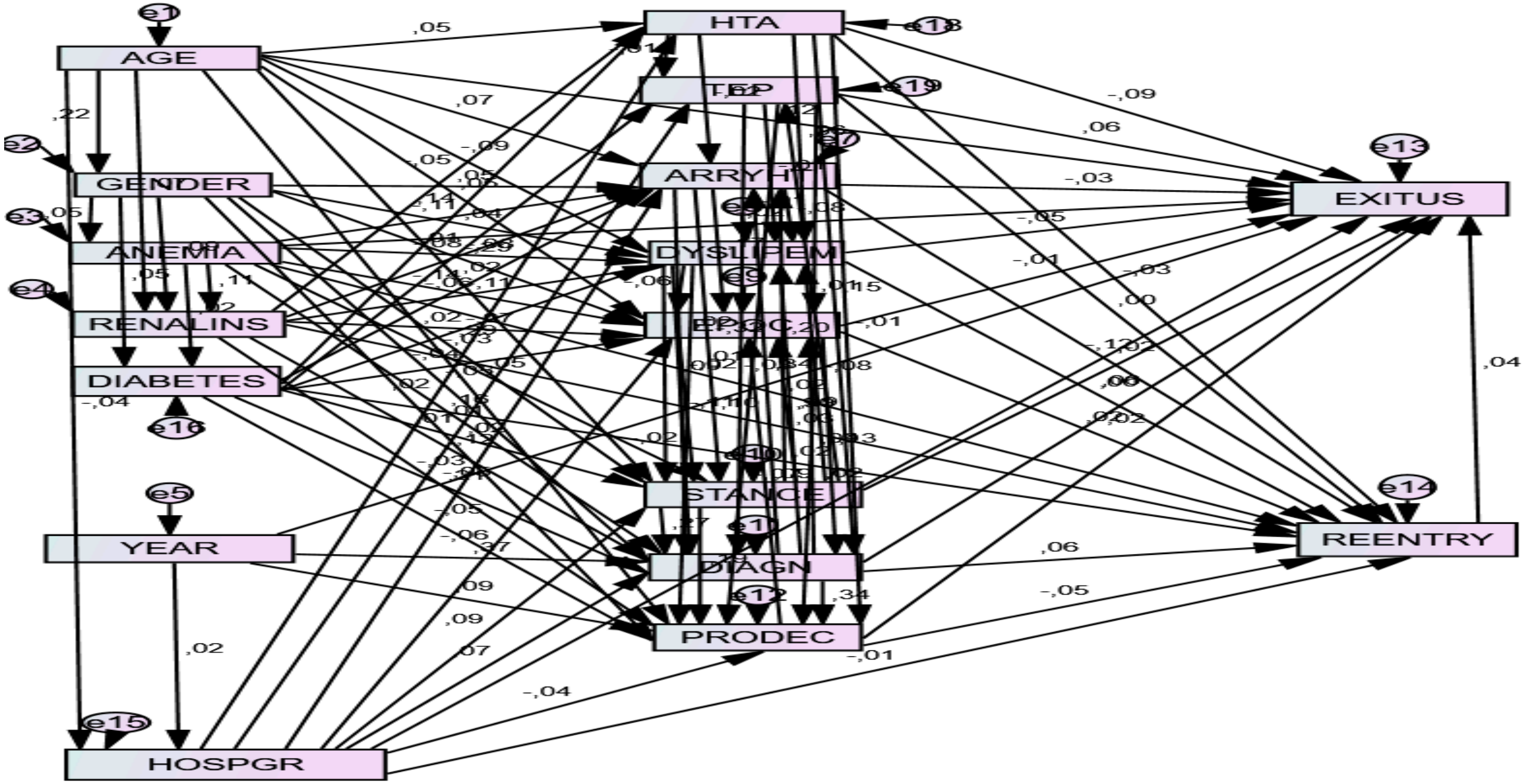

Model S2. ADVANCED SECOND-TIER MODEL FOR HOSPITAL MORTALITY .

Estimates (Group number 1 - Default model)

Scalar Estimates (Group number 1 - Default model)

Maximum Likelihood Estimates

Regression Weights: (Group number 1 - Default model)

			Estimate	S.E.	C.R.	P	Label
D3	<---	D1	0.000	0.000	14.589	***	
D2	<---	D1	0.051	0.001	43.618	***	
D2	<---	D3	65.057	2.962	21.961	***	
D4	<---	D3	66.542	3.050	21.819	***	
D5	<---	D4	5.280	0.090	58.792	***	
D5	<---	D1	-0.179	0.004	-41.592	***	
D5	<---	D2	0.512	0.036	14.238	***	
D6	<---	D1	0.044	0.007	6.249	***	
D6	<---	D5	0.499	0.038	12.983	***	
D6	<---	D2	-0.168	0.024	-6.876	***	
D6	<---	D3	-202.756	21.001	-9.655	***	
REENTRY	<---	D6	0.130	0.003	38.831	***	
EXITUS	<---	D6	0.247	0.005	51.443	***	
GENDER	<---	D1	1.000				
AGE	<---	D1	4.441	0.061	72.608	***	
DIABETES	<---	D2	1.000				
DYSLIPEM	<---	D2	0.685	0.004	179.532	***	
HTA	<---	D2	0.970	0.006	174.341	***	
ARRYHT	<---	D4	1.000				
YEAR	<---	D4	9.604	0.053	182.470	***	
TEP	<---	D3	1.000				
HOSPGR	<---	D3	64.458	2.892	22.285	***	
EPOC	<---	D1	-0.228	0.003	-74.535	***	
PRODEC	<---	D5	1.000				
STANCE	<---	D5	1.070	0.008	136.147	***	
RENALINS	<---	D6	1.000				
ANEMIA	<---	D5	0.091	0.001	161.636	***	

			Estimate	S.E.	C.R.	P	Label
DIAGN	<---	D5	4.089	0.021	190.635	***	

Standardized Regression Weights: (Group number 1 - Default model)

			Estimate
D3	<---	D1	0.060
D2	<---	D1	0.136
D2	<---	D3	0.582
D4	<---	D3	0.921
D5	<---	D4	0.870
D5	<---	D1	-0.122
D5	<---	D2	0.131
D6	<---	D1	0.103
D6	<---	D5	1.713
D6	<---	D2	-0.147
D6	<---	D3	-1.587
REENTRY	<---	D6	0.087
EXITUS	<---	D6	0.170
GENDER	<---	D1	0.974
AGE	<---	D1	0.223
DIABETES	<---	D2	0.391
DYSLIPEM	<---	D2	0.372
HTA	<---	D2	0.352
ARRYHT	<---	D4	0.234
YEAR	<---	D4	0.358
TEP	<---	D3	0.027
HOSPGR	<---	D3	0.104
EPOC	<---	D1	-0.282
PRODEC	<---	D5	0.319
STANCE	<---	D5	0.157
RENALINS	<---	D6	0.599
ANEMIA	<---	D5	0.195
DIAGN	<---	D5	1.069

Intercepts: (Group number 1 - Default model)

	Estimate	S.E.	C.R.	P	Label
RENALINS	0.139	0.000	353.972	***	
REENTRY	0.107	0.000	304.402	***	
EXITUS	0.101	0.000	295.479	***	
GENDER	1.559	0.001	2765.445	***	
AGE	76.890	0.011	7031.039	***	
EPOC	0.187	0.000	422.678	***	
DIABETES	0.312	0.001	593.175	***	
DYSLIPEM	0.127	0.000	336.633	***	
HTA	0.475	0.001	837.587	***	
ARRYHT	0.516	0.001	908.657	***	
YEAR	2002.420	0.004	561550.168	***	
DIAGN	6.527	0.003	2113.396	***	
PRODEC	1.789	0.003	707.187	***	
STANCE	8.003	0.005	1458.957	***	
TEP	0.004	0.000	53.319	***	
HOSPGR	2.465	0.001	2163.368	***	
ANEMIA	0.125	0.000	332.819	***	

Variances: (Group number 1 - Default model)

	Estimate	S.E.	C.R.	P	Label
e18	0.234	0.003	77.045	***	
e20	0.000	0.000	11.205	***	
e19	0.021	0.000	68.748	***	
e21	0.002	0.000	9.715	***	
e22	0.053	0.004	12.214	***	
e23	0.013	0.003	4.612	***	
e16	0.095	0.000	614.876	***	
e17	0.088	0.000	581.498	***	
e1	0.013	0.003	4.229	***	
e2	88.131	0.154	573.892	***	
e3	0.140	0.000	511.172	***	
e4	0.182	0.000	514.773	***	
e5	0.096	0.000	528.893	***	
e6	0.218	0.000	542.134	***	
e10	0.236	0.000	581.163	***	
e9	8.595	0.019	445.624	***	

	Estimate	S.E.	C.R.	P	Label
e11	-1.049	0.034	-31.098	***	
e13	4.460	0.007	602.377	***	
e12	22.755	0.037	623.179	***	
e7	0.004	0.000	622.641	***	
e8	0.995	0.002	616.435	***	
e15	0.077	0.001	90.543	***	
e14	0.105	0.000	622.084	***	

Matrices (Group number 1 - Default model)

Implied Covariances (Group number 1 - Default model)

	ANEMIA	RENALINS	HOSPGR	TEP	STANCE	PRODEC	DIAGN	YEAR	ARRYHT	HTA	DYSLIPEM	DIABETES	EPOC	AGE	GENDER	EXITUS	REENTRY
ANEMIA	0.109																
RENALINS	0.003	0.120															
HOSPGR	0.006	-0.004	1.006														
TEP	0.000	0.000	0.000	0.004													
STANCE	0.049	0.038	0.069	0.001	23.333												
PRODEC	0.046	0.035	0.065	0.001	0.540	4.965											
DIAGN	0.187	0.144	0.265	0.004	2.210	2.065	7.396										
YEAR	0.068	0.015	0.108	0.002	0.800	0.747	3.055	9.861									
ARRYHT	0.007	0.002	0.011	0.000	0.083	0.078	0.318	0.132	0.250								
HTA	0.007	-0.002	0.011	0.000	0.078	0.073	0.298	0.107	0.011	0.249							
DYSLIPEM	0.005	-0.002	0.008	0.000	0.055	0.051	0.210	0.076	0.008	0.022	0.111						
DIABETES	0.007	-0.003	0.011	0.000	0.080	0.075	0.307	0.111	0.012	0.032	0.022	0.215					
EPOC	0.000	0.002	-0.001	0.000	0.004	0.004	0.016	-0.007	-0.001	-0.003	-0.002	-0.003	0.152				
AGE	-0.007	-0.047	0.013	0.000	-0.084	-0.079	-0.321	0.133	0.014	0.064	0.045	0.066	-0.237	92.742			
GENDER	-0.002	-0.011	0.003	0.000	-0.019	-0.018	-0.072	0.030	0.003	0.015	0.010	0.015	-0.053	1.038	0.247		
EXITUS	0.001	0.011	-0.001	0.000	0.009	0.009	0.036	0.004	0.000	-0.001	0.000	-0.001	0.001	-0.012	-0.003	0.091	
REENTRY	0.000	0.006	0.000	0.000	0.005	0.005	0.019	0.002	0.000	0.000	0.000	0.000	0.000	-0.006	-0.001	0.001	0.095

Implied Correlations (Group number 1 - Default model)

	ANEMIA	RENALINS	HOSPGR	TEP	STANCE	PRODEC	DIAGN	YEAR	ARRYHT	HTA	DYSLIPEM	DIABETES	EPOC	AGE	GENDER	EXITUS	REENTRY
ANEMIA	1.000																
RENALINS	0.028	1.000															
HOSPGR	0.018	-0.011	1.000														
TEP	0.005	-0.003	0.003	1.000													
STANCE	0.031	0.023	0.014	0.004	1.000												
PRODEC	0.062	0.046	0.029	0.007	0.050	1.000											
DIAGN	0.208	0.153	0.097	0.025	0.168	0.341	1.000										
YEAR	0.065	0.014	0.034	0.009	0.053	0.107	0.358	1.000									
ARRYHT	0.043	0.009	0.022	0.006	0.034	0.070	0.234	0.084	1.000								
HTA	0.040	-0.014	0.022	0.006	0.032	0.065	0.219	0.069	0.045	1.000							
DYSLIPEM	0.042	-0.015	0.023	0.006	0.034	0.069	0.232	0.072	0.047	0.131	1.000						
DIABETES	0.044	-0.016	0.024	0.006	0.036	0.073	0.244	0.076	0.050	0.138	0.145	1.000					
EPOC	0.003	0.018	-0.002	0.000	0.002	0.005	0.016	-0.006	-0.004	-0.017	-0.018	-0.019	1.000				
AGE	-0.002	-0.014	0.001	0.000	-0.002	-0.004	-0.012	0.004	0.003	0.013	0.014	0.015	-0.063	1.000			
GENDER	-0.010	-0.061	0.006	0.002	-0.008	-0.016	-0.054	0.019	0.013	0.059	0.062	0.065	-0.275	0.217	1.000		
EXITUS	0.008	0.102	-0.003	-0.001	0.006	0.013	0.043	0.004	0.003	-0.004	-0.004	-0.004	0.005	-0.004	-0.017	1.000	
REENTRY	0.004	0.052	-0.002	0.000	0.003	0.007	0.022	0.002	0.001	-0.002	-0.002	-0.002	0.003	-0.002	-0.009	0.015	1.000

Implied Means (Group number 1 - Default model)

	ANEMIA	RENALINS	HOSPGR	TEP	STANCE	PRODEC	DIAGN	YEAR	ARRYHT	HTA	DYSLIPEM	DIABETES	EPOC	AGE	GENDER	EXITUS	REENTRY
	0.125	0.139	2.465	0.004	8.003	1.789	6.527	2002.420	0.516	0.475	0.127	0.312	0.187	76.890	1.559	0.101	0.107

Total Effects (Group number 1 - Default model)

	D1	D3	D4	D2	D5	D6
D3	0.000	0.000	0.000	0.000	0.000	0.000
D4	0.013	66.542	0.000	0.000	0.000	0.000
D2	0.064	65.057	0.000	0.000	0.000	0.000

	D1	D3	D4	D2	D5	D6
D5	-0.076	384.656	5.280	0.512	0.000	0.000
D6	-0.045	-21.640	2.636	0.088	0.499	0.000
ANEMIA	-0.007	34.878	0.479	0.046	0.091	0.000
RENALINS	-0.045	-21.640	2.636	0.088	0.499	1.000
HOSPGR	0.013	64.458	0.000	0.000	0.000	0.000
TEP	0.000	1.000	0.000	0.000	0.000	0.000
STANCE	-0.081	411.557	5.649	0.548	1.070	0.000
PRODEC	-0.076	384.656	5.280	0.512	1.000	0.000
DIAGN	-0.309	1572.762	21.588	2.094	4.089	0.000
YEAR	0.128	639.091	9.604	0.000	0.000	0.000
ARRYHT	0.013	66.542	1.000	0.000	0.000	0.000
HTA	0.062	63.109	0.000	0.970	0.000	0.000
DYSLIPEM	0.044	44.566	0.000	0.685	0.000	0.000
DIABETES	0.064	65.057	0.000	1.000	0.000	0.000
EPOC	-0.228	0.000	0.000	0.000	0.000	0.000
AGE	4.441	0.000	0.000	0.000	0.000	0.000
GENDER	1.000	0.000	0.000	0.000	0.000	0.000
EXITUS	-0.011	-5.341	0.651	0.022	0.123	0.247
REENTRY	-0.006	-2.821	0.344	0.011	0.065	0.130

Standardized Total Effects (Group number 1 - Default model)

	D1	D3	D4	D2	D5	D6
D3	0.060	0.000	0.000	.000	0.000	0.000
D4	0.055	0.921	0.000	.000	0.000	0.000
D2	0.171	0.582	0.000	.000	0.000	0.000
D5	-0.051	0.877	0.870	.131	0.000	0.000
D6	-0.105	-0.169	1.491	.077	1.713	0.000
ANEMIA	-0.010	0.171	0.170	.025	0.195	0.000
RENALINS	-0.063	-0.101	0.892	.046	1.025	0.599
HOSPGR	0.006	0.104	0.000	.000	0.000	0.000
TEP	0.002	0.027	0.000	.000	0.000	0.000
STANCE	-0.008	0.138	0.137	.021	0.157	0.000
PRODEC	-0.016	0.280	0.278	.042	0.319	0.000
DIAGN	-0.055	0.938	0.930	.140	1.069	0.000
YEAR	0.020	0.330	0.358	.000	0.000	0.000
ARRYHT	0.013	0.216	0.234	.000	0.000	0.000

	D1	D3	D4	D2	D5	D6
HTA	0.060	0.205	0.000	.352	0.000	0.000
DYSLIPEM	0.064	0.217	0.000	.372	0.000	0.000
DIABETES	0.067	0.228	0.000	.391	0.000	0.000
EPOC	-0.282	0.000	0.000	.000	0.000	0.000
AGE	0.223	0.000	0.000	.000	0.000	0.000
GENDER	0.974	0.000	0.000	.000	0.000	0.000
EXITUS	-0.018	-0.029	0.253	.013	0.290	0.170
REENTRY	-0.009	-0.015	0.130	.007	0.150	0.087

Direct Effects (Group number 1 - Default model)

	D1	D3	D4	D2	D5	D6
D3	0.000	0.000	0.000	0.000	0.000	0.000
D4	0.000	66.542	0.000	0.000	0.000	0.000
D2	0.051	65.057	0.000	0.000	0.000	0.000
D5	-0.179	0.000	5.280	0.512	0.000	0.000
D6	0.044	-202.756	0.000	-0.168	0.499	0.000
ANEMIA	0.000	0.000	0.000	0.000	0.091	0.000
RENALINS	0.000	0.000	0.000	0.000	0.000	1.000
HOSPGR	0.000	64.458	0.000	0.000	0.000	0.000
TEP	0.000	1.000	0.000	0.000	0.000	0.000
STANCE	0.000	0.000	0.000	0.000	1.070	0.000
PRODEC	0.000	0.000	0.000	0.000	1.000	0.000
DIAGN	0.000	0.000	0.000	0.000	4.089	0.000
YEAR	0.000	0.000	9.604	0.000	0.000	0.000
ARRYHT	0.000	0.000	1.000	0.000	0.000	0.000
HTA	0.000	0.000	0.000	0.970	0.000	0.000
DYSLIPEM	0.000	0.000	0.000	0.685	0.000	0.000
DIABETES	0.000	0.000	0.000	1.000	0.000	0.000
EPOC	-0.228	0.000	0.000	0.000	0.000	0.000
AGE	4.441	0.000	0.000	0.000	0.000	0.000
GENDER	1.000	0.000	0.000	0.000	0.000	0.000
EXITUS	0.000	0.000	0.000	0.000	0.000	0.247
REENTRY	0.000	0.000	0.000	0.000	0.000	0.130

Standardized Direct Effects (Group number 1 - Default model)

	D1	D3	D4	D2	D5	D6
D3	0.060	0.000	0.000	0.000	0.000	0.000
D4	0.000	0.921	0.000	0.000	0.000	0.000
D2	0.136	0.582	0.000	0.000	0.000	0.000
D5	-0.122	0.000	0.870	0.131	0.000	0.000
D6	0.103	-1.587	0.000	-0.147	1.713	0.000
ANEMIA	0.000	0.000	0.000	0.000	0.195	0.000
RENALINS	0.000	0.000	0.000	0.000	0.000	0.599
HOSPGR	0.000	0.104	0.000	0.000	0.000	0.000
TEP	0.000	0.027	0.000	0.000	0.000	0.000
STANCE	0.000	0.000	0.000	0.000	0.157	0.000
PRODEC	0.000	0.000	0.000	0.000	0.319	0.000
DIAGN	0.000	0.000	0.000	0.000	1.069	0.000
YEAR	0.000	0.000	0.358	0.000	0.000	0.000
ARRYHT	0.000	0.000	0.234	0.000	0.000	0.000
HTA	0.000	0.000	0.000	0.352	0.000	0.000
DYSLIPEM	0.000	0.000	0.000	0.372	0.000	0.000
DIABETES	0.000	0.000	0.000	0.391	0.000	0.000
EPOC	-0.282	0.000	0.000	0.000	0.000	0.000
AGE	0.223	0.000	0.000	0.000	0.000	0.000
GENDER	0.974	0.000	0.000	0.000	0.000	0.000
EXITUS	0.000	0.000	0.000	0.000	0.000	0.170
REENTRY	0.000	0.000	0.000	0.000	0.000	0.087

Indirect Effects (Group number 1 - Default model)

	D1	D3	D4	D2	D5	D6
D3	0.000	0.000	.000	0.000	0.000	0.000
D4	0.013	0.000	0.000	0.000	0.000	0.000
D2	0.013	0.000	0.000	0.000	0.000	0.000
D5	0.103	384.656	0.000	0.000	0.000	0.000
D6	-0.089	181.116	2.636	0.256	0.000	0.000
ANEMIA	-0.007	34.878	0.479	0.046	0.000	0.000
RENALINS	-0.045	-21.640	2.636	0.088	0.499	0.000
HOSPGR	0.013	0.000	0.000	0.000	0.000	0.000
TEP	0.000	0.000	0.000	0.000	0.000	0.000
STANCE	-0.081	411.557	5.649	0.548	0.000	0.000
PRODEC	-0.076	384.656	5.280	0.512	0.000	0.000

	D1	D3	D4	D2	D5	D6
DIAGN	-0.309	1572.762	21.588	2.094	0.000	0.000
YEAR	0.128	639.091	0.000	0.000	0.000	0.000
ARRYHT	0.013	66.542	0.000	0.000	0.000	0.000
HTA	0.062	63.109	0.000	0.000	0.000	0.000
DYSLIPEM	0.044	44.566	0.000	0.000	0.000	0.000
DIABETES	0.064	65.057	0.000	0.000	0.000	0.000
EPOC	0.000	0.000	0.000	0.000	0.000	0.000
AGE	0.000	0.000	0.000	0.000	0.000	0.000
GENDER	0.000	0.000	0.000	0.000	0.000	0.000
EXITUS	-0.011	-5.341	0.651	0.022	0.123	0.000
REENTRY	-0.006	-2.821	0.344	0.011	0.065	0.000

Standardized Indirect Effects (Group number 1 - Default model)

	D1	D3	D4	D2	D5	D6
D3	0.000	0.000	0.000	0.000	0.000	0.000
D4	0.055	0.000	0.000	0.000	0.000	0.000
D2	0.035	0.000	0.000	0.000	0.000	0.000
D5	0.070	0.877	0.000	0.000	0.000	0.000
D6	-0.208	1.417	1.491	0.224	0.000	0.000
ANEMIA	-0.010	0.171	0.170	0.025	0.000	0.000
RENALINS	-0.063	-0.101	0.892	0.046	1.025	0.000
HOSPGR	0.006	0.000	0.000	0.000	0.000	0.000
TEP	0.002	0.000	0.000	0.000	0.000	0.000
STANCE	-0.008	0.138	0.137	0.021	0.000	0.000
PRODEC	-0.016	0.280	0.278	0.042	0.000	0.000
DIAGN	-0.055	0.938	0.930	0.140	0.000	0.000
YEAR	0.020	0.330	0.000	0.000	0.000	0.000
ARRYHT	0.013	0.216	0.000	0.000	0.000	0.000
HTA	0.060	0.205	0.000	0.000	0.000	0.000
DYSLIPEM	0.064	0.217	0.000	0.000	0.000	0.000
DIABETES	0.067	.228	0.000	0.000	0.000	0.000
EPOC	0.000	.000	0.000	0.000	0.000	0.000
AGE	0.000	.000	0.000	0.000	0.000	0.000
GENDER	0.000	.000	0.000	0.000	0.000	0.000
EXITUS	-0.018	-.029	0.253	0.013	0.290	0.000
REENTRY	-0.009	-.015	0.130	0.007	0.150	0.000

Model Fit Summary

CMIN

Model	NPAR	CMIN	DF	P	CMIN/DF
Default model	62	208062.711	108	0.000	1926.507
Saturated model	170	0.000	0		
Independence model	17	820419.358	153	0.000	5362.218

Baseline Comparisons

Model	NFI Delta1	RFI rho1	IFI Delta2	TLI rho2	CFI
Default model	0.946	0.941	0.946	0.941	0.946
Saturated model	1.000		1.000		1.000
Independence model	0.000	0.000	.000	0.000	0.000

Parsimony-Adjusted Measures

Model	PRATIO	PNFI	PCFI
Default model	0.706	0.527	0.527
Saturated model	0.000	0.000	0.000
Independence model	1.000	0.000	0.000

NCP

Model	NCP	LO 90	HI 90
Default model	207954.711	206457.481	209458.215
Saturated model	0.000	0.000	0.000
Independence model	820266.358	817289.917	823249.074

FMIN

Model	FMIN	F0	LO 90	HI 90
Default model	0.268	0.268	0.266	0.270
Saturated model	0.000	0.000	0.000	0.000

Model	FMIN	F0	LO 90	HI 90
Independence model	1.058	1.058	1.054	1.062

RMSEA

Model	RMSEA	LO 90	HI 90	PCLOSE
Default model	0.050	0.050	0.050	0.941
Independence model	0.083	0.083	0.083	0.000

AIC

Model	AIC	BCC	BIC	CAIC
Default model	208186.711	208186.713		
Saturated model	340.000	340.008		
Independence model	820453.358	820453.359		

ECVI

Model	ECVI	LO 90	HI 90	MECVI
Default model	0.268	0.267	0.270	0.268
Saturated model	0.000	0.000	0.000	0.000
Independence model	1.058	1.054	1.062	1.058

HOELTER

Model	HOELTER 0.05	HOELTER 0.01
Default model	497	541
Independence model	173	186
